# Investigation into the use of histone deacetylase inhibitor MS-275 as a topical agent for the prevention and treatment of cutaneous squamous cell carcinoma in an SKH-1 hairless mouse model

**DOI:** 10.1371/journal.pone.0213095

**Published:** 2019-03-13

**Authors:** Jay H. Kalin, Abdulkerim Eroglu, Hua Liu, W. David Holtzclaw, Irene Leigh, Charlotte M. Proby, Jed W. Fahey, Philip A. Cole, Albena T. Dinkova-Kostova

**Affiliations:** 1 Department of Medicine, Division of Genetics, Brigham and Women’s Hospital, Boston, MA, United States of America; 2 Department of Biological Chemistry and Molecular Pharmacology, Harvard Medical School, Boston, MA, United States of America; 3 Department of Pharmacology and Molecular Sciences, Johns Hopkins University School of Medicine, Baltimore, MD, United States of America; 4 Cullman Chemoprotection Center, Johns Hopkins University, Baltimore, MD, United States of America; 5 Division of Cancer Research, Jacqui Wood Cancer Centre, University of Dundee, Dundee, United Kingdom; 6 Department of Medicine, Johns Hopkins University School of Medicine, Baltimore, MD, United States of America; 7 Department of International Health, Johns Hopkins University Bloomberg School of Public Health, Baltimore, MD, United States of America; Ohio State University, UNITED STATES

## Abstract

Cutaneous squamous cell carcinomas are a common form of highly mutated keratinocyte skin cancers that are of particular concern in immunocompromised patients. Here we report on the efficacy of topically applied MS-275, a clinically used histone deacetylase inhibitor, for the treatment and management of this disease. At 2 mg/kg, MS-275 significantly decreased tumor burden in an SKH-1 hairless mouse model of UVB radiation-induced skin carcinogenesis. MS-275 was cell permeable as a topical formulation and induced histone acetylation changes in mouse tumor tissue. MS-275 was also effective at inhibiting the proliferation of patient derived cutaneous squamous cell carcinoma lines and was particularly potent toward cells isolated from a regional metastasis on an immunocompromised individual. Our findings support the use of alternative routes of administration for histone deacetylase inhibitors in the treatment of high-risk squamous cell carcinoma which may ultimately lead to more precise delivery and reduced systemic toxicity.

## Introduction

Cutaneous squamous cell carcinoma (cSCC) is the second most commonly diagnosed keratinocyte skin cancer with an incidence of 15–35 per 100,000 and is increasing at a rate of 2–4% each year [1]. The mortality rate for metastatic cSCC has been reported to be as high as 70% [2] and accounts for 20% of all skin cancer related deaths [3]. Although various surgical techniques, radiotherapy, and chemotherapy are often effective against primary tumors, 3–5% of them recur and can metastasize anywhere in the body [2,4]. Organ transplant recipients and other patients receiving immunosuppressive drugs are exceptionally vulnerable with a 65 to 100-fold greater incidence and more than a 25-fold higher rate of metastasis [5,6]. In addition, cSCC and keratoacanthomas develop rapidly (within several weeks of treatment) in approximately 15 to 30% of patients treated with inhibitors of the oncogenic serine/threonine protein kinase BRAF, such as vemurafenib and dabrafenib [7,8].

Ultraviolet radiation is a ubiquitous, complete carcinogen (an initiator and a promoter) and it is the main etiological factor associated with the development of cSCC [9]. UV exposure, particularly UVB (280–315 nm), stimulates production of immunosuppressive cytokines, leads to direct DNA damage, and results in generation of reactive oxygen species that damage biomolecules including DNA [5]. As such, these cSCC tumors often carry a heavy mutational burden [10] with tumor suppressor genes being some of the most frequently mutated genes in both mice and humans [11–13]. The immunosuppressive drug azathioprine exacerbates susceptibility to DNA damage [14] since its metabolites are incorporated into DNA and contribute to the generation of reactive oxygen species upon UV irradiation, damaging DNA and proteins, including those involved in DNA repair [15,16]. Thus, new approaches to mitigate the risk of cSCC development and progression in high-risk immunocompromised patients would be of significant clinical benefit.

**Fig 1:**
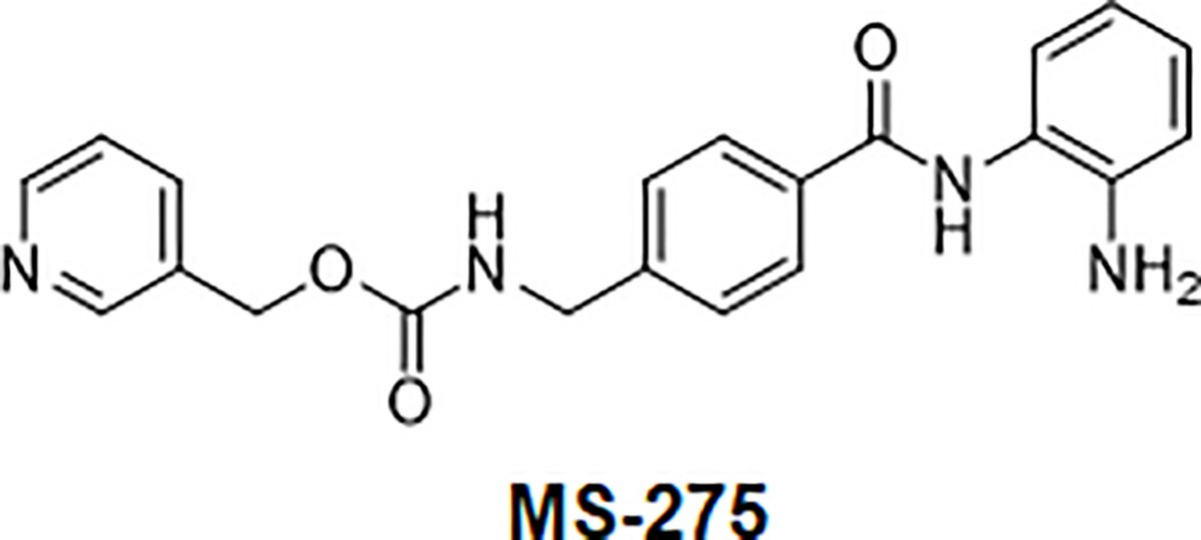
Chemical structure of MS-275 (entinostat).

Histone deacetylase inhibitors (HDACi) are already clinically used for the treatment of T-cell lymphoma and multiple myeloma and are being further evaluated in combination with other chemotherapeutic and biologic medications [17]. More recently, HDACi were shown to be effective anticancer agents by inducing the expression of tumor suppressor genes in melanoma mouse xenograft models [18,19] and cSCC cell lines [18]. Herein we report the topical administration of a class I selective HDACi, MS-275 (Entinostat, Fig 1), as a chemopreventive compound in a mouse model of UVB-radiation induced cSCC. HDACi anticancer activity is typically derived through inhibition of the class I isoforms, so selective compounds should alleviate toxicity arising through broad spectrum HDAC inhibition [20]. In addition, benzamide compounds like MS-275 exhibit slow, tight-binding pharmacodynamics toward HDAC1-3 [21] and have long half-lives in vivo [22] which may allow for lower, less frequent dosing. Furthermore, local (topical) HDACi application may be therapeutically effective at lower concentrations, ultimately lessening the side effect profile associated with systemic administration [23,24]. We demonstrate that topical delivery of MS-275 to the skin of SKH-1 hairless mice inhibits tumor growth and upregulates histone acetylation in tumor tissue at doses below 2 mg/kg. We also show that MS-275 inhibits the proliferation of patient derived primary and metastatic cSCC cell lines at low- to sub-micromolar concentrations and induces expression of the tumor suppressor p21. Taken together, our results suggest that topically administered MS-275 may be a viable strategy for treating cSCC in high-risk populations.

## Results

**Fig 2:**
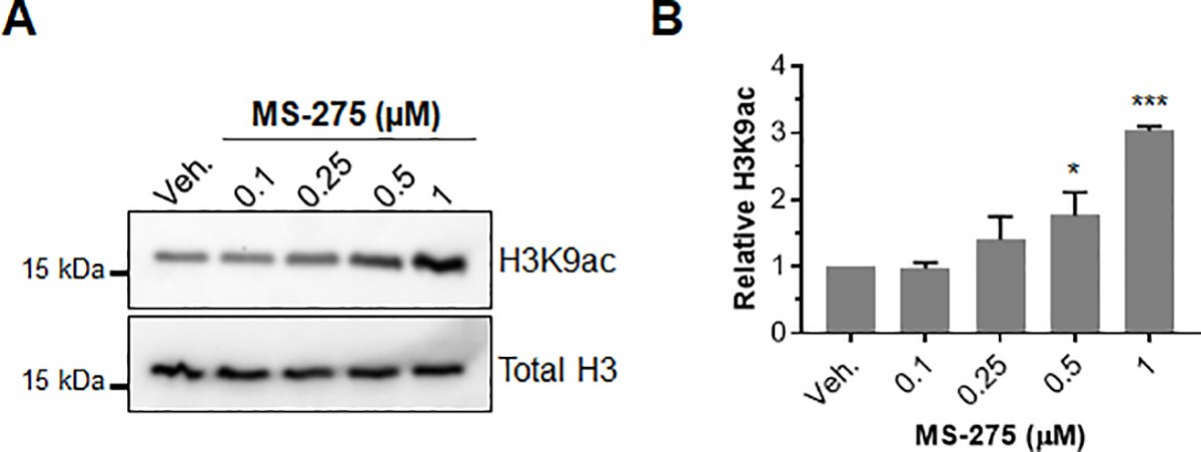
The effect of MS-275 on histone acetylation in mouse Kera-308 cells. (**A**) Representative Western blots depicting dose dependent increases in histone H3K9 acetylation upon treatment with the indicated amount of drug for 24 h. H3K9ac and total H3 blots were run separately on different gels using aliquots from the same sample preparations and processed in parallel. Uncropped Western blots are available as S5A Fig. (**B**) H3K9 acetylation levels were normalized to total H3 and quantified by densitometry using ImageJ (n = 3, unpaired *t* test, *p < 0.05, ***p < 0.001).

cSCCs are of keratinocyte origin. Thus, we first evaluated the ability of MS-275 to induce histone acetylation in cultured mouse Kera-308 keratinocytes. In our previous study, we found that histone H3 lysine 9 acetylation (H3K9ac), along with H3K4 methylation and H3K18 acetylation, were consistently induced by MS-275 and other class I HDACi across different skin cancer cell lines [18]. Due to the robust nature of the H3K9ac increase, we concluded that it could serve as a representative biomarker for class I HDAC inhibition and transcriptional activation in vivo [18,25]. In Kera-308 cells, dose response studies indicated that modest induction of histone acetylation was achieved at 250 nM and that robust, 3-fold increases occurred with 1 μM treatment after 24 h exposure (Fig 2A and 2B).

To evaluate the effect of topical MS-275 treatment on UVB radiation-induced skin carcinogenesis, beginning at 8-weeks of age, mice were exposed twice weekly to 30 mJ/cm^2^ UVB radiation per session for 18 weeks. Considering that the UVB radiation-induced skin carcinogenesis experiments typically last between 30 and 40 weeks, we wanted to use the minimally effective dose of inhibitor to reduce potential side effects associated with long term treatment [26]. Thus, starting at week 1 and continuing throughout the study, two groups of 30 mice each were treated with either 0.1 μmol MS-275 or an 80% acetone/water vehicle, topically across their dorsal skin, three times a week on days that they were not exposed to UV radiation. Of note, the acetone/water vehicle was chosen because it evaporates quickly and limits the removal of compound through grooming. It has been used previously in mice and humans without any deleterious effects and may help to permeabilize the stratum corneum [27–29].

**Fig 3:**
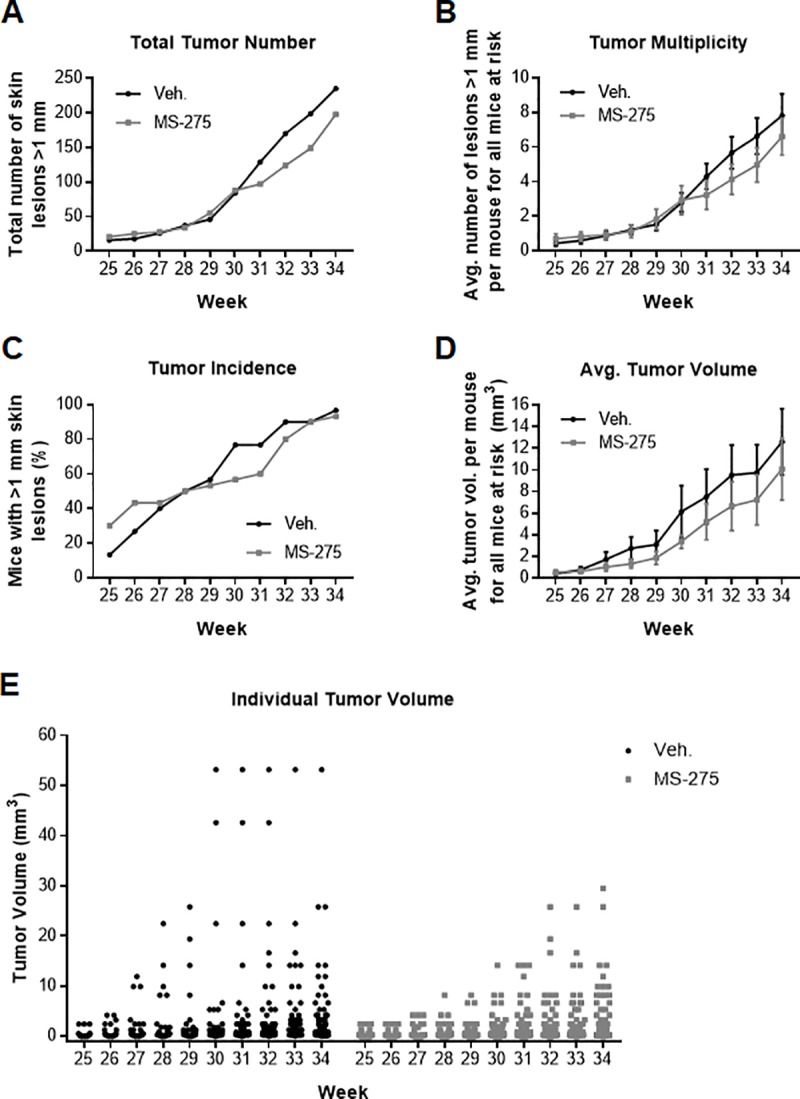
Analysis of MS-275 efficacy when applied topically in an SKH-1 hairless mouse model of UVB-induced cutaneous squamous cell carcinoma. (**A**) Total number of tumors per group by week for ten weeks as defined by lesions ≥ 1 mm in diameter (n = 30 mice per group). (**B**) Tumor multiplicity for each group by week as defined by the average number of tumors for all mice at risk. (**C**) Tumor incidence by week as defined by the percentage of mice with lesions. (**D**) Tumor burden as defined by the average tumor volume per mouse for all mice at risk. (**E**) Individual tumor volumes plotted by week and by group.

Tumor development was monitored for 10 weeks, beginning at study week 25, and was continued until 95% of the animals in one group had developed tumors (study week 34) which were defined as lesions >1 mm in diameter on the dorsal skin. At the time of sacrifice, there were a total of 235 tumors present on the vehicle treated animals and 198 tumors on the MS-275 treated animals (Fig 3A). Of note, one animal in the vehicle treated group had a large tumor which likely broke off on week 29 and was consequently censored for this analysis. However, no significant differences were observed with regard to tumor multiplicity as defined by the average number of tumors per animal (Fig 3B). Additionally, by the end of the study, there was no significant difference in tumor incidence between the two groups as nearly all of the mice had developed tumors (vehicle group, 29/30 mice; MS-275 group, 28/30 mice, Fig 3C).

In contrast to tumor multiplicity and incidence, the average tumor burden (volume, expressed in mm^3^) was significantly affected by MS-275 treatment compared to vehicle control (Fig 3D). Regression analysis indicated that tumors grew more slowly in the treatment group (F = 113.99, *p* < 0.00005) and that MS-275 significantly decreased average tumor volume relative to vehicle in this model (*p* = 0.003). In addition, although not robustly significant, individual tumor burden in the treatment group was smaller compared to the control group (Fig 3E, *p* = 0.082). In other words, tumors on the vehicle treated mice were generally larger and therefore likely more advanced in stage relative to the inhibitor treated group. No significant differences in mouse body weight trajectories were observed between the two groups throughout the study indicating that MS-275 treatment was well tolerated (S1 Fig). Animal behavior was also stable with no signs of abnormal food or water consumption, changes in grooming, or locomotion.

**Fig 4:**
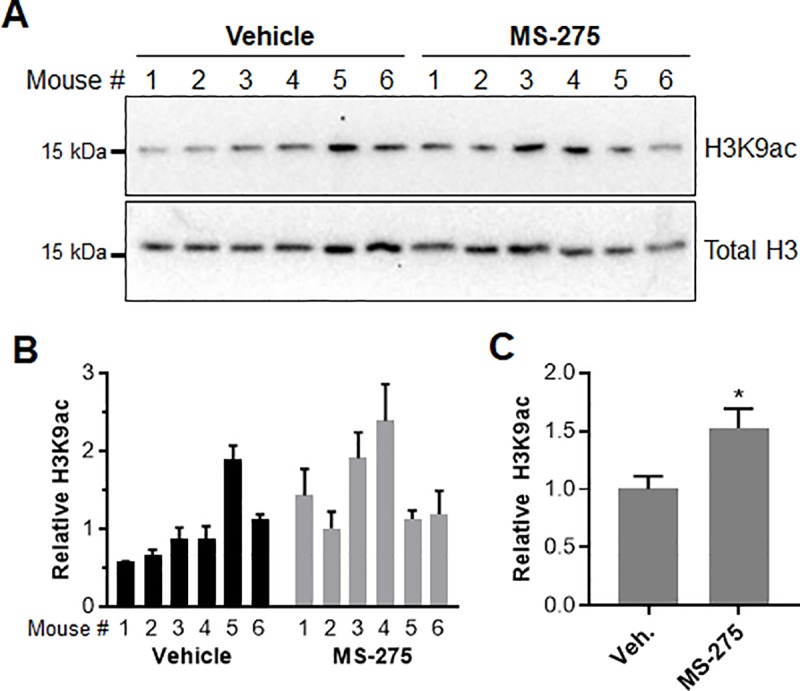
Analysis of tumor tissue obtained from mice at the end of the cutaneous carcinogenesis study. (**A**) Representative Western blots showing increases in H3K9 acetylation in tumors treated with MS-275 as compared to vehicle. Tumor tissue was selected at random from 6 mice in each group (1 mouse from each cage). Mice were sacrificed and tissue was isolated 12–16 h after the last inhibitor dose. H3K9ac and total H3 blots were run separately on different gels using aliquots from the same sample preparations and processed in parallel. Uncropped Western blots are available as S5B Fig. (**B**) H3K9 acetylation levels were normalized to total H3 and quantified by densitometry using ImageJ (n = 3). (**C**) Average increase in histone H3K9 acetylation for MS-275 and vehicle treated groups (n = 6, unpaired *t* test, *p < 0.05).

Upon conclusion of the study, mice were euthanized 12–16 h after their last treatment. One mouse from each cage was chosen at random (resulting in a total of six mice per group), and used for isolation of tumor tissue and analysis of histone acetylation changes by Western blot (Fig 4A). To control for pharmacokinetic variation, tumors of similar size (1–2 mm^3^) were selected for analysis. In most cases, multiple tumors were needed to obtain sufficient amounts of tissue. H3K9ac levels were quantified using densitometry and normalized to total H3 levels in tumor tissue homogenates (Fig 4B). On average, histone H3K9 acetylation increased 1.5-fold in the tumor tissue of MS-275 treated animals relative to control (Fig 4C). This modest increase in histone acetylation is consistent with the low dose used in this study and the amount of time (12 to 16 h) that elapsed between the final treatment and sacrifice of the mice.

**Fig 5:**
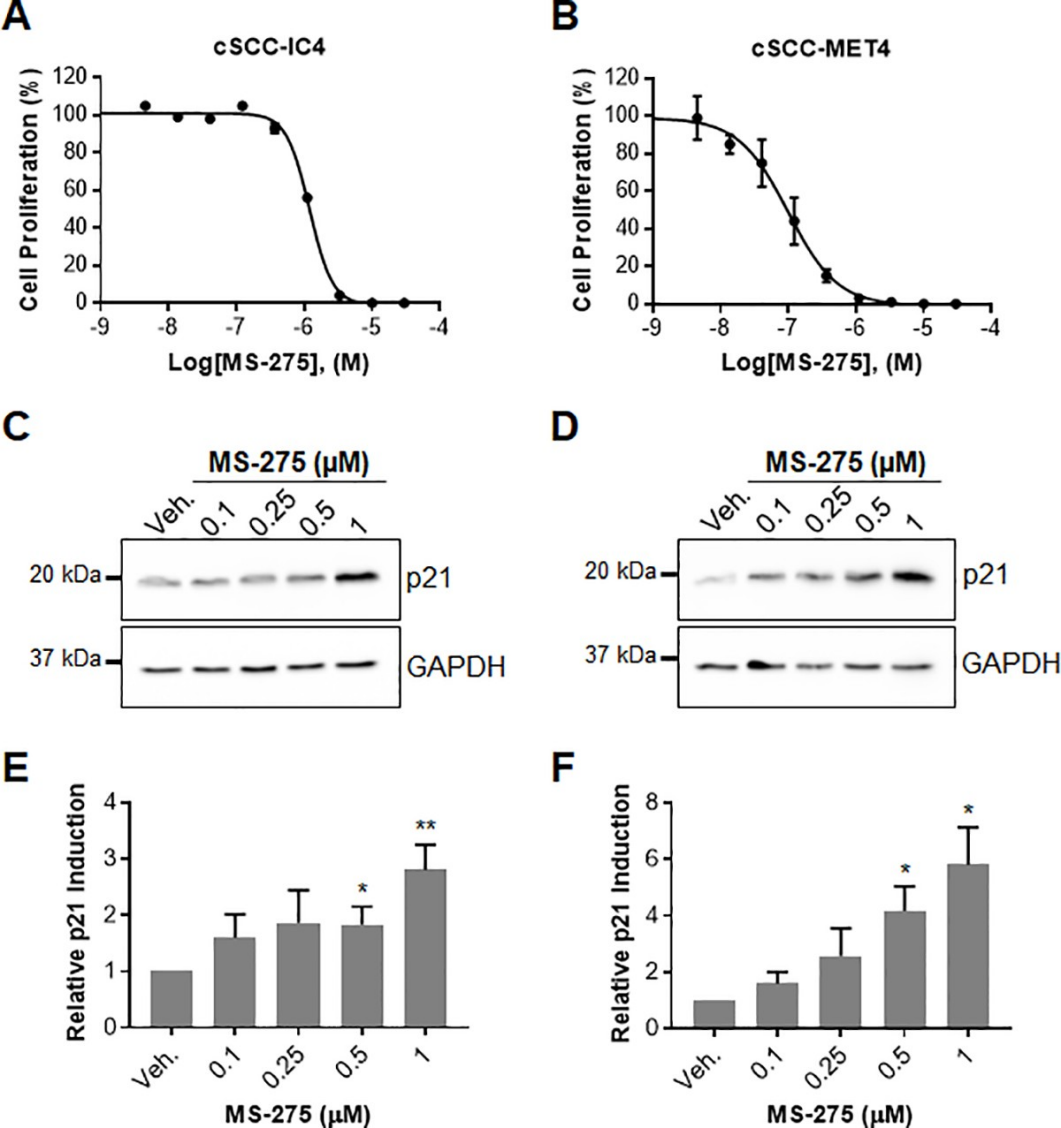
The effect of MS-275 on the proliferation of patient derived cutaneous squamous cell carcinoma cell lines. (**A**) cSCC-IC4 (IC_50_ = 1.24 ± 0.05 μM) and (**B**) cSCC-MET4 (IC_50_ = 0.10 ± 0.02 μM) cells were seeded in 96-well plates for 24 h prior to addition of the indicated amount of inhibitor in DMSO (final concentration of DMSO was 0.5%). Cells were cultured for an additional 72 h with [^3^H]thymidine being added 6 h prior to harvesting cells. Radioactivity was measured using a Microbeta scintillation counter and normalized to vehicle treated controls (IC_50_ = mean ± SE, n = 4). Representative Western blots in (**C**) cSCC-IC4 and (**D**) cSCC-MET4 cell lines depicting dose dependent increases in p21 levels upon treatment with the indicated amount of drug for 24 h. GAPDH and p21 were detected in samples that were run on the same gel. After transfer to a nitrocellulose membrane, the membrane was cut at the 25 kDa marker and membrane sections were incubated with the indicated primary antibody. Quantitation of p21 levels by densitometry for (**E**) cSCC-IC4 and (**F**) cSCC-MET4 cell lines using ImageJ (n = 3, unpaired *t* test, *p < 0.05, **p < 0.01).

Lastly, to determine if the beneficial effects observed with MS-275 in murine cSCCs would translate to humans, we evaluated the ability of this compound to inhibit the proliferation of three primary human cSCC cell lines. cSCC-IC4 and cSCC-IC8 cells were isolated from immunocompetent patients whereas the cSCC-MET4 cell line was established from a regional metastasis in a renal transplant recipient [30]. MS-275 inhibited the proliferation of both IC4 and IC8 cell lines with IC_50_ values of 1.24 ± 0.05 μM and 0.74 ± 0.09 μM, respectively (Fig 5A, S2 Fig). Importantly, MS-275 potently inhibited proliferation of the MET4 metastatic cell line from the immunocompromised individual with an IC_50_ of 0.10 ± 0.02 μM (Fig 5B). These data are in close agreement with our previous findings in related cell lines [18]. Briefly, cSCC-MET1 cells isolated from the same immunocompromised patient’s primary tumor were much more sensitive to the anti-proliferative effect of MS-275 with an IC_50_ of 0.14 ± 0.01 μM compared to cSCC-IC1 cells isolated from a primary tumor on an immunocompetent individual (IC1 cells, IC_50_ = 1.03 ± 0.07 μM).

In our previous study, we identified 153 tumor suppressor genes that were induced by 2.5 μM MS-275 treatment in melanoma cells and found that p21 mRNA expression was upregulated by more than 20-fold [18]. Here, we show that 500 nM MS-275 was sufficient to significantly increase p21 levels, with 3- and 6-fold increases being observed at 1 μM in IC4 and MET4 cells, respectively (Fig 5C–5F). Interestingly, baseline p21 levels were 8-fold lower in MET4 relative to IC4 cells (S3 Fig). In addition, we evaluated p21 levels in skin tumor tissue from our mouse study by qRT-PCR, however, we did not observe any differences between vehicle and MS-275 treated samples (S4 Fig). One possible explanation is that the 12–16 h time difference between the last dose and tissue collection was too long. At this time point, a subtle cumulative effect may be difficult to detect. Alternatively, p21 expression may have been upregulated in the tumor-free areas of the skin, but not in the tumors themselves. Lastly, given the very long (>30 week) duration of the study, it is also possible that the transcriptional regulatory machinery became desensitized to MS-275 treatment over time, resulting in a diminished effect of the drug by the time samples were collected. Loss of p21 regulation is often observed in cancer [31] and, although additional studies are needed to optimize p21 detection in mouse tissue, our data indicate that the efficacy of MS-275 in cSCC may be related to its ability to restore the expression of p21 and other tumor suppressor genes. Taken together, these results suggest that the use of MS-275 could be a potential strategy for treating cSCC in high-risk immunocompromised populations.

## Discussion

cSCC continues to represent an unmet medical need in immunocompromised patients who are considered to be at high-risk for disease development. While primary tumors are typically treatable, immunocompromised individuals are at a much greater risk for recurrence and metastasis for which there are fewer available treatment options. Here, we showed that the HDACi MS-275 was found to be beneficial in both a mouse model of cSCC as well as in patient derived cell lines. HDACi are used clinically to treat hematologic malignancies, but significant side effect profiles have limited their application to broader areas of oncology [32]. However, the topical route of administration used here could limit systemic circulation of the drug and allow for a lower effective concentration [23].

In this study, mice treated topically with approximately 2 mg/kg MS-275 presented with significantly decreased average tumor volume suggesting that MS-275 could effectively slow tumor growth at this dose. In addition, although not statistically significant by conventional standards (*p* < 0.05), the individual tumor volume, total number of tumors, and tumor multiplicity were all lower in the MS-275 treated animals. While the antitumor effect of MS-275 was modest in this pilot study, higher doses are achievable with this formulation and would likely improve efficacy. However, additional studies will be needed to fully characterize dose dependent systemic exposure and establish an optimal dosing regimen. Studies comparing the efficacy of topically administered MS-275 in treatment and management models as opposed to the chemoprevention model used here would also help determine the best course of action for therapeutic intervention.

In addition to optimizing treatment conditions, investigating the mechanism of action of HDACi in cSCC may lead to the discovery of alternative treatment strategies. A common characteristic of cSCC cancers in both mice and humans is that they typically present with a high mutational burden and their mutational spectrum is dominated by mutations in tumor suppressor genes [11,12,18]. Furthermore, exposure to UV radiation, the main causative factor in cSCC, is itself immunosuppressive and further promotes disease progression [5]. Here, we showed that MS-275 can induce expression of the tumor suppressor p21 in human cSCC cells, however, the precise mechanism of action for its anticancer effect remains unknown. Identification of other gene expression changes induced by MS-275 in cSCCs may reveal alternative targets downstream of HDAC where pharmacological intervention could be beneficial. HDACs are also known to play an important role in regulating antigen presentation [33] and immune response, suggesting that additional studies are warranted to clearly identify the relevant pathways as well as the particular HDAC isoforms and complexes involved [34,35].

In summary, our results show that the HDACi MS-275 can be delivered topically and is effective at slowing tumor growth in mice. In primary human cSCC cell lines, MS-275 inhibited cell proliferation at concentrations in the low- to sub-micromolar range, and was particularly potent in cells isolated from an immunocompromised patient. While our data suggest that MS-275 could be beneficial in these high-risk patients, screening of additional cell lines, especially from immunosuppressed individuals, would be needed to firmly establish this trend. Although additional work is necessary to optimize the route of administration, together our data support the idea that HDACi can be used effectively as topical agents in the treatment and management of cSCC.

## Methods

### Cell culture

Cells were maintained in a 5% CO_2_ atmosphere at 37°C and confirmed to be free of mycoplasma contamination prior to initiating experiments. Mouse Kera-308 cells were purchased from Cell Line Services (Eppelheim, Germany) and cultured in DMEM supplemented with 10% FBS. cSCC-IC4 and cSCC-IC8 cultures were established from primary tumors on the shin of a 73-year-old female immunocompetent patient and the buttock of a 51-year-old female immunocompetent patient, respectively. The cSCC-MET4 cell line was established from a regional metastasis in the left axillary lymph node of a 56-year-old male renal transplant recipient. cSCC cell lines were cultured in a 3:1 mixture of DMEM (high glucose, + L-glutamine)/F12 Nutrient Mixture supplemented with 10% FBS, 40 μg/mL hydrocortisone, 500 μg/mL insulin, 1 μg/mL EGF, 0.84 μg/mL cholera toxin, 500 μg/mL transferrin, and 1.3 μg/mL liothyronine.

### Histone extraction and Western blot

Cells were cultured to 70% confluency and then treated with inhibitor for 24 h. After treatment, cells were collected in cold PBS and pelleted at 300 x *g*. Tumor tissue samples were isolated as described below. Histones were extracted as previously described [36]. Briefly, cells were homogenized/resuspeneded in cold hypotonic lysis buffer (10 mM Tris-HCl pH 8.0, 1 mM KCl, 1.5 mM MgCl_2_, 1 mM DTT, and 1X Roche EDTA-free cOmplete Protease Inhibitor Cocktail) and incubated on a rotator at 4°C for 30 min. Nuclei were pelleted by spinning at 10,000 x *g* for 10 min at 4°C and the supernatant was discarded. Nuclei were resuspended in 400 μL of 0.4 N H_2_SO_4_ and incubated on a rotator at 4°C for either 30 min (cell culture) or 1 h (tumor tissue samples). Solutions were clarified by spinning at 16,000 x *g* for 10 min after which the supernatant was transferred to a new Eppendorf tube. Additional spins were necessary to completely remove debris from tumor tissue samples. Once clarified, histones were precipitated by adding 132 μL TCA (100% w/v). Samples were incubated on ice for 30 min and then histones were pelleted by centrifugation at 16,000 x *g* for 10 min at 4°C. Histones were washed twice with 250 μL of cold acetone and then allowed to dry overnight. After drying, histones were resuspended in 200 μL of water and protein concentration was determined using the micro BCA assay (ThermoFisher Scientific). Proteins were resolved on 15% SDS-gels and then transferred to nitrocellulose membranes. Membranes were blocked with 5% BSA in TBST for 1 h at room temperature and then incubated with H3K9ac (Abcam, ab4441, 1:1000) or total H3 (Abcam, ab1791, 1:5000) primary antibody overnight at 4°C. After washing with TBST, membranes were incubated with HRP-conjugated secondary antibody (Cell Signaling Technologies, 7074S, 1:5000), washed again with TBST, and then imaged using Amersham ECL Western Blotting Detection Reagents (GE Healthcare) and a Syngene PXi imaging system. Information regarding antibody characterization is available through the Manufacturer’s website. Band quantitation by densitometry was done with ImageJ and the unpaired *t* test was used to determine statistical significance.

### Whole cell lysate and Western blot

Cells were cultured to 70% confluency and then treated with inhibitor for 24 h. After treatment, cells were collected in cold PBS and pelleted at 300 x *g*. Cells were resuspended in RIPA buffer (MilliporeSigma) supplemented with 5X Roche EDTA-free cOmplete Protease Inhibitor Cocktail and incubated on a rotator at 4°C for 30 min. Solutions were clarified by spinning at 16,000 x *g* for 10 min after which the supernatant was transferred to a new Eppendorf tube and protein concentration was determined using the micro BCA assay (ThermoFisher Scientific). Proteins were resolved on 15% SDS-gels and Western blots were carried out as described above, but substituting Clarity Western ECL Substrate (Bio-Rad) as the imaging reagent. Primary antibodies: p21 (Cell Signaling, 2947, 1:2000), GAPDH (Cell Signaling, 2118S, 1:5000). Information regarding antibody characterization is available through the Manufacturer’s website. Band quantitation by densitometry was done with ImageJ and the unpaired *t* test was used to determine statistical significance.

### [^3^H]Thymidine incorporation assay

cSCC cells were seeded in 96-well plates (2500 cells/well for IC4 and IC8, 1250 cells/well for MET4) and cultured for 24 h prior to addition of inhibitor. Cells were then treated for an additional 72 h with the indicated concentration of drug (vehicle = DMSO, 0.5% final concentration). At the 66 h time point, 10 μL of a 0.1 mCi/mL solution of tritiated thymidine (American Radiolabeled Chemicals) was added. Cells were harvested using a Filtermate Harvester (PerkinElmer) and radioactivity was measured using a Microbeta liquid scintillation counter (PerkinElmer). Data represent at least two independent experiments with four technical replicates per experiment.

### UVB radiation-induced skin carcinogenesis experiment

All animal work was approved by and done in accordance with the Johns Hopkins University Institutional Animal Care and Use Committee (protocol number MO14M299). Female SKH-1 hairless mice (Charles River) were housed with 35% humidity using a 12 h light/dark cycle and free access to food and water. Cutaneous carcinogenesis was initiated when the mice were 8-weeks old by subjecting the animals chronically twice a week (Tuesday and Thursday) for 18 weeks to UV radiation (30 mJ/cm^2^ UVB per session). The radiant dose was quantified with a UVB Daavlin Flex Control Integrating Dosimeter and further confirmed using an external X-96 Irradiance Meter (Daavlin) before and after each irradiation session. The mice were placed in clear, bedding-free cages prior to UV radiation exposure. The animals were kept within their social groups during all irradiation sessions, and an electrical fan was used to minimize any possibility of discomfort due to heat from the lamps.

Two groups of 30 mice each (5 mice per cage) were treated topically on their dorsal skin with MS-275 (0.1 μmol in 100 μL of 80% acetone/water) or vehicle alone three times per week (Monday, Wednesday, Friday) for the duration of the experiment. Tumor development was monitored for 10 weeks, beginning at study week 25. Tumors (defined as lesions >1 mm in diameter) that formed on the dorsal skin were measured and mapped once a week. The mice were weighed and examined regularly throughout the experiment to ensure compliance with humane and scientific endpoints. To minimize suffering, no tumors were allowed to grow larger than 1 cm in diameter, the total tumor burden did not exceed 10% of body weight, and the animals were observed carefully for any signs of discomfort as judged by their behavior, food consumption, and weight gain. Tumor volume was calculated as 4/3πr^3^ with the radius derived from the average diameter as measured in three dimensions (length, width, height). To determine statistical significance, regression analysis was carried out using the Statistics/Data Analysis package in Stata v.11.2 (StataCorp LP). At termination of the experiment, the animals were euthanized by terminal anesthesia using halothane, and tumor tissue was isolated, flash frozen, and stored at -80°C.

### qRT-PCR

Randomly chosen frozen tumor tissue from each mouse (one mouse per cage resulting in a total of six samples per group) was pulverized and homogenized in Buffer RLT from the RNeasy Mini Kit (Qiagen). Total RNA from each homogenate was then extracted following the RNeasy Mini Kit (Qiagen) protocol. cDNA was synthesized using the iScript cDNA Synthesis Kit (Bio-Rad Laboratories). qRT-PCR analysis was performed using the QuantStudio 3 Real-Time PCR System (Applied Biosystems). All primers were optimized, and a final primer concentration of 300 nM was used for all reactions. Primer sequences for gene amplification were as follows: p21, forward 5′-caaagtgtgccgttgtctctt-3′, reverse 5′-tcaaagttccaccgttctcg-3′; and GAPDH (endogenous control), forward 5′-tcaacggcacagtcaagg-3′, reverse 5′-actccacgacatactcagc-3′. The reactions were assembled using 2.5 ng of cDNA, 1 x PowerUp SYBR Green Master Mix (Applied Biosystems), forward and reverse primers, and nuclease-free water. Relative mRNA expression was normalized to GAPDH. Gene expression was calculated using the comparative 2^−ΔΔCT^ method [37].

## Supporting information

S1 Fig The effect of topically applied MS-275 (0.1 μmol) on mouse body weight. Mice were weighed once per week during the last ten weeks of the study. Data are reported as mean ± SE (n = 30 mice per group).
(TIF)

S2 Fig The effect of MS-275 on the proliferation of patient derived cutaneous squamous cell carcinoma cell line cSCC-IC8. IC_50_ = 0.74 ± 0.09 μM. Cells were seeded in 96-well plates for 24 h prior to addition of the indicated amount of inhibitor in DMSO (final concentration of DMSO was 0.5%). Cells were cultured for an additional 72 h with [^3^H]thymidine being added 6 h prior to harvesting cells. Radioactivity was measured using a Microbeta scintillation counter and normalized to vehicle treated controls (IC_50_ = mean ± SE, n = 4).
(TIF)

S3 Fig Evaluation of baseline p21 levels in cSCC-IC4 and cSCC-MET4 cell lines. (**A**) Representative Western blots comparing p21 levels in vehicle treated (0.5% DMSO) cSCC-IC4 and cSCC-MET4 cell lines. GAPDH and p21 were detected in samples that were run on the same gel. After transfer to a nitrocellulose membrane, the membrane was cut at the 25 kDa marker and membrane sections were incubated with the indicated primary antibody. (**B**) p21 levels were normalized to GAPDH and quantified by densitometry using ImageJ (n = 3, unpaired *t* test, **p < 0.01). (**C**) Uncropped Western blots from S3A Fig. Prestained ladder was imaged separately and overlaid with Western blot.
(TIF)

S4 Fig Analysis of p21 levels in mouse tumor tissue. (**A**) p21 mRNA levels were quantified by qRT-PCR and normalized to GAPDH (n = 3). (**B**) Average change in p21 levels for MS-275 and vehicle treated groups (n = 6).
(TIF)

S5 Fig Uncropped Western blots. (**A**) Fig 2A. (**B**) Fig 4A. (**C**) Fig 5C. (**D**) Fig 5D. Prestained ladder was imaged separately and overlaid with Western blot.
(TIF)

S1 Data Raw data used to generate manuscript figures. (XLSX)
