# Supplementary figures and images for: Investigation into the use of histone deacetylase inhibitor MS-275 as a topical agent for the prevention and treatment of cutaneous squamous cell carcinoma in an SKH-1 hairless mouse model
Source: PLoS One. 2019 Mar 13;14(3):e0213095. doi: 10.1371/journal.pone.0213095 (PMC6415858; doi:10.1371/journal.pone.0213095)

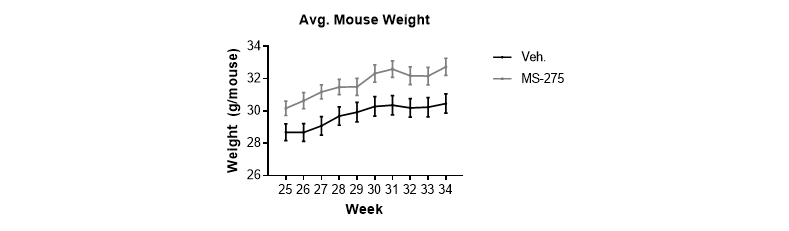

Supplement: S1 Fig — Mice were weighed once per week during the last ten weeks of the study. Data are reported as mean ± SE (n = 30 mice per group). (TIF) [file pone.0213095.s001.tif]

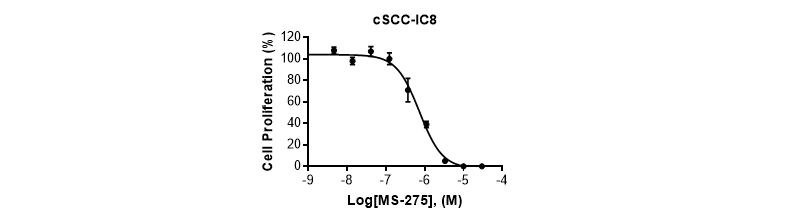

Supplement: S2 Fig — IC50 = 0.74 ± 0.09 μM. Cells were seeded in 96-well plates for 24 h prior to addition of the indicated amount of inhibitor in DMSO (final concentration of DMSO was 0.5%). Cells were cultured for an additional 72 h with [3H]thymidine being added 6 h prior to harvesting cells. Radioactivity was measured using a Microbeta scintillation counter and normalized to vehicle treated controls (IC50 = mean ± SE, n = 4). (TIF) [file pone.0213095.s002.tif]

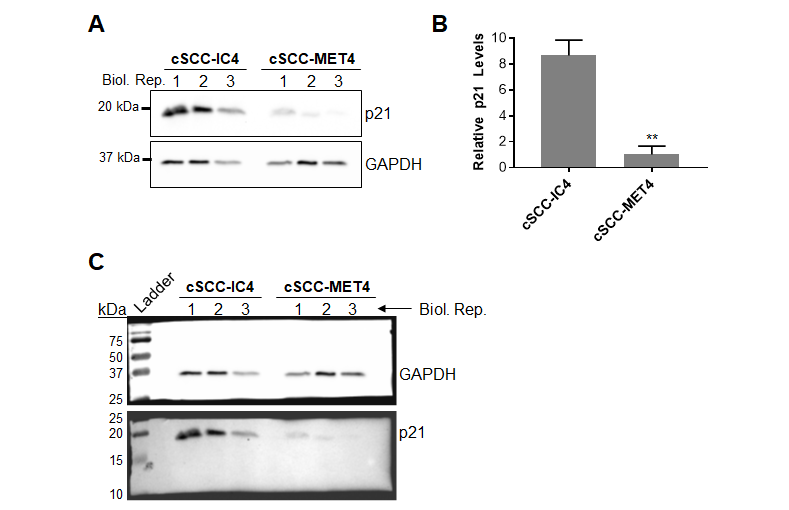

Supplement: S3 Fig — (A) Representative Western blots comparing p21 levels in vehicle treated (0.5% DMSO) cSCC-IC4 and cSCC-MET4 cell lines. GAPDH and p21 were detected in samples that were run on the same gel. After transfer to a nitrocellulose membrane, the membrane was cut at the 25 kDa marker and membrane sections were incubated with the indicated primary antibody. (B) p21 levels were normalized to GAPDH and quantified by densitometry using ImageJ (n = 3, unpaired t test, **p < 0.01). (C) Uncropped Western blots from S3A Fig. Prestained ladder was imaged separately and overlaid with Western blot. (TIF) [file pone.0213095.s003.tif]

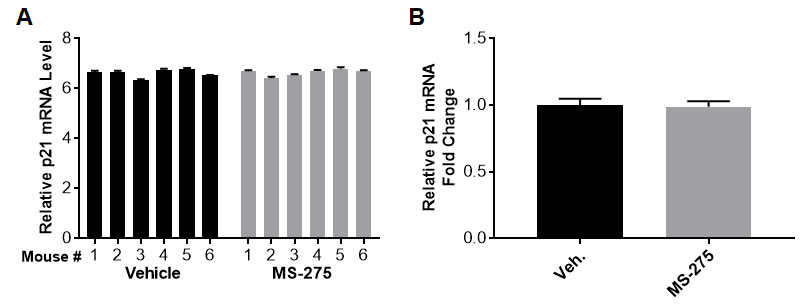

Supplement: S4 Fig — (A) p21 mRNA levels were quantified by qRT-PCR and normalized to GAPDH (n = 3). (B) Average change in p21 levels for MS-275 and vehicle treated groups (n = 6). (TIF) [file pone.0213095.s004.tif]

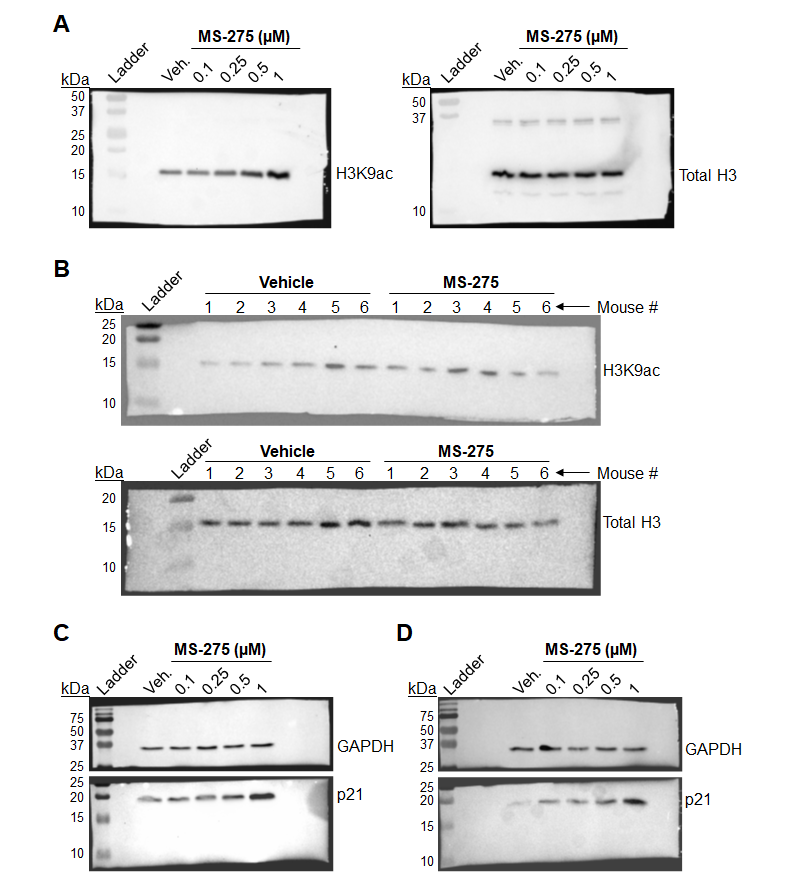

Supplement: S5 Fig — (A) Fig 2A. (B) Fig 4A. (C) Fig 5C. (D) Fig 5D. Prestained ladder was imaged separately and overlaid with Western blot. (TIF) [file pone.0213095.s005.tif]
